# Multiple etiologies of axonal sensory motor polyneuropathy in a renal transplant recipient: a case report

**DOI:** 10.1186/1752-1947-5-530

**Published:** 2011-10-27

**Authors:** Jalal Etemadi, Mohammadali M Shoja, Kamyar Ghabili, Mahnaz Talebi, Hossein Namdar, Reshad Mirnour

**Affiliations:** 1 Department of Nephrology, Dialysis and Transplantation, Tabriz University of Medical Sciences, Tabriz, Iran; 2 Medical Philosophy and History Research Center, Tabriz University of Medical Sciences, Tabriz, Iran; 3 Tuberculosis and Lung Disease Research Center, Tabriz University of Medical Sciences, Tabriz, Iran; 4 Neuroscience Research Center, Tabriz University of Medical Sciences, Tabriz, Iran; 5 Department of Cardiology, Tabriz University of Medical Sciences, Tabriz, Iran

## Abstract

**Introduction:**

Neurological complications leading to morbidity and mortality are not frequent in renal transplant recipients. Here, we report a renal transplant recipient who presented with diminished strength in his limbs probably due to multiple etiologies of axonal sensorimotor polyneuropathy, which resolved with intravenous immunoglobulin.

**Case presentation:**

A 49-year-old Iranian male renal transplant recipient with previous history of autosomal dominant polycystic kidney disease presented with diminished strength in his limbs one month after surgery. Our patient was on cyclosporine A, mycophenolate mofetil and prednisone. Although a detected hypophosphatemia was corrected with supplemental phosphate, the loss of strength was still slowly progressive and diffuse muscular atrophy was remarkable in his trunk, upper limb and pelvic girdle. Meanwhile, his cranial nerves were intact. Post-transplant diabetes mellitus was diagnosed and insulin therapy was initiated. In addition, as a high serum cyclosporine level was detected, the dose of cyclosporine was reduced. Our patient was also put on intravenous ganciclovir due to positive serum cytomegalovirus immunoglobulin M antibody. Despite the reduction of oral cyclosporine dose along with medical therapy for the cytomegalovirus infection and diabetes mellitus, his muscular weakness and atrophy did not improve. One week after administration of intravenous immunoglobulin, a significant improvement was noted in his muscular weakness.

**Conclusion:**

A remarkable response to intravenous immunoglobulin is compatible with an immunological basis for the present condition (post-transplant polyneuropathy). In cases of post-transplant polyneuropathy with a high clinical suspicion of immunological origin, administration of intravenous immunoglobulin may be recommended.

## Introduction

Widespread and symmetric dysfunction of peripheral nerves, known as polyneuropathy, may follow a number of medical conditions (such as diabetes mellitus and nutritional deficiency), toxic exposures (drug or environmental) and infectious agents (for example, cytomegalovirus (CMV) infection) [1]. Neurological complications such as polyneuropathies leading to morbidity and mortality are not frequent in renal transplant recipients [2,3]. Here, we report a renal transplant recipient who presented with diminished strength in his limbs, probably due to multiple etiologies of axonal sensorimotor polyneuropathy, which resolved with intravenous immunoglobulin (IVIG).

## Case report

A 49-year-old male Iranian patient underwent living unrelated renal transplantation after two years of hemodialysis. The cause of his renal failure was autosomal dominant polycystic kidney disease. Both donor and recipient were seronegative for CMV. The initial postoperative period was uneventful and our patient was discharged on a regimen of cyclosporine A, mycophenolate mofetil, prednisone, isoniazid and vitamin B6 (pyridoxine). One month later, he noticed diminished strength in his arms and legs. At that time, laboratory tests revealed hypophosphatemia. Therefore, supplemental phosphate was started. However, his loss of strength was still slowly progressive. Two months later, he was unable to get up from a chair and lift his arms over his head. Neurologic examinations revealed diffuse muscular atrophy in his trunk, shoulders, upper limbs, thenar eminence and pelvic girdle. His deep tendon reflexes were symmetrically diminished. The muscle force grading was 3/5 in his proximal upper limbs, 4/5 in his distal upper limbs, 2/5 in his proximal lower limbs and 4/5 in his distal lower limbs. Sensory examinations were normal and his cranial nerves were intact.

Laboratory tests revealed hemoglobin, 13.9 g/dL; platelet count, 77 × 10^3^/mm^3^; creatinine, 1.2 mg/dL; urea, 25 mg/dL; sodium, 137 mmol/L; potassium, 4.8 mmol/L; calcium, 9 mg/dL; phosphorus, 2.4 mg/dL; serum parathyroid hormone, 180 pg/mL (normal range, 17.3 to 72.9 pg/mL); aldolase, 3 U/mL (normal range: 0 to 7.6 U/mL); creatine phosphokinase, 41 IU/L (normal range: 24 to 170 IU/L); and lactate dehydrogenase, 1053 IU/L (normal range up to 480 IU/L). Moreover, his blood glucose level was elevated (400 mg/dL) and urine analysis showed glucosuria (3+); therefore, post-transplant diabetes mellitus was diagnosed and insulin therapy was commenced. Electromyography with a nerve conduction study revealed severe axonal dominant sensory motor polyneuropathy. However, his serum cyclosporine level was 872 ng/mL (normal range, 250 to 375 ng/mL); therefore, the dose of oral cyclosporine was reduced from 225 to 150 mg twice daily. His serum CMV immunoglobulin M (IgM) antibody was positive; therefore intravenous ganciclovir (5 mg/kg/day) was administrated [4,5]. A lumbar puncture to analyze cerebrospinal fluid was not feasible due to thrombocytopenia.

**Figure 1:**
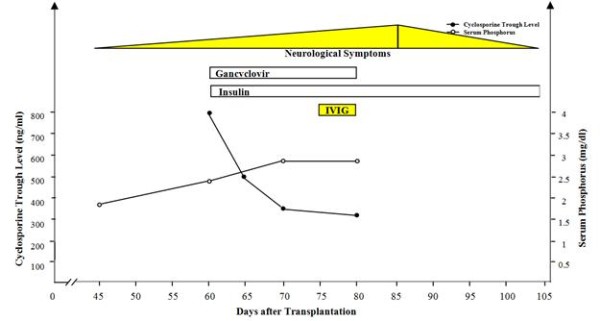
**Trend of neurological symptoms, serum cyclosporine and phosphorus levels and treatment protocols during the post-transplant period**.

Fifty days after the reduction of the cyclosporine dose and medical therapy for both CMV infection and post-transplant diabetes mellitus, our patient's muscular atrophy and weakness had not improved. IVIG was commenced (400 mg/kg/day) for five days. One week later, significant improvement was noted in his muscular weakness. Figure 1 illustrates the trend of neurological symptoms, serum cyclosporine and phosphorus levels and treatment protocols during the post-transplant period in our patient.

## Discussion

Cyclosporine toxicity may induce a wide range of neurological disorders both in the central nervous system and peripheral nerves [6,7]. Neurological complications are usually reversible after dose reduction or discontinuation of the medical therapy, although there have been cases of permanent or even fatal damage [8]. Guarino *et al. *observed cyclosporine-induced motor polyneuropathy in four out of nineteen patients after liver transplantation. Two patients had demyelinating and axonal damage and all the patients recovered within two months of cyclosporine cessation [9]. Terrovitis *et al. *reported cyclosporine-associated axonal degenerative symmetric polyneuropathy in a patient one month after cardiac transplantation [6]. In the present case, cyclosporine toxicity might have contributed to the development of subacute axonal polyneuropathy. Hence, cyclosporine-induced polyneuropathy should be considered in patients with neurological complications following kidney transplantation.

Our patient developed diabetes mellitus two months after the renal transplantation. Diabetes mellitus is commonly implicated in the development of the neuropathy. A remarkable number of patients with non-insulin dependent diabetes mellitus are affected by slowly progressive and irreversible neuropathy [10]. Nonetheless, since the pattern of the polyneuropathy in the present case was both acute and reversible, diabetes mellitus seemed not to be an etiological factor.

CMV is also commonly associated with polyneuropathy [11]. Identified in 10% to 15% of patients with Guillian-Barré syndrome (GBS), CMV infection is the most common antecedent viral disease [12]. In a case report, CMV polyneuropathy presented in two kidney transplant recipients as GBS [13]. In our patient, CMV infection, clinical manifestations and electromyography and nerve conduction study findings were all in favor of a GBS diagnosis.

Our patient was on isoniazid as a prophylactic treatment of tuberculosis; renal transplantation increases the risk of tuberculosis infection [14]. One of the earliest known side effects of isoniazid is peripheral neuropathy characterized by paresthesia and weakness. All symptoms usually disappear following isoniazid withdrawal. Pyridoxine can prevent the neurological side effects of isoniazid [15]. In the presented case, symptoms disappeared while our patient was still on isoniazid prophylaxis therapy. Moreover, pyridoxine was already given to prevent peripheral neuropathy. Thus, isoniazid-induced neurological syndrome seems not to be the etiology of the polyneuropathy in our patient.

Hypophosphatemia, a serum phosphate level of less than 2.5 mg/dL [16], can cause a wide range of disorders including central nervous system disorders and peripheral neuropathy. The latter can present with areflexia and muscle weakness. These symptoms have been reported in cases of hypophosphatemia [17,18]. Hypophosphatemia was detected in our patient as well. However, the clinical presentations of polyneuropathy did not improve following correction of this electrolyte disturbance. As a result, the polyneuropathy does not seem to be attributable to hypophosphatemia in our patient.

We found the present case noteworthy as more than one etiology can be considered for the peripheral neuropathy presented in our patient. These etiologies include cyclosporine neurotoxicity, CMV-induced polyneuropathy, diabetes mellitus, isoniazid-induced neuropathy and hypophosphatemia. Since the symptoms significantly improved once IVIG was administered, CMV-induced polyneuropathy or GBS seems to be the most probable cause of muscle weakness in the present report.

## Conclusion

A remarkable response to IVIG is compatible with an immunological basis for the present condition (in other words, post-transplant polyneuropathy). In cases of post-transplant polyneuropathy with a high clinical suspicion of the immunological origin (such as CMV-induced polyneuropathy or GBS), administration of IVIG may be recommended.

## Abbreviations

CMV: cytomegalovirus; GBS: Guillian-Barré syndrome; IVIG: intravenous immunoglobulin.

## Consent

Written informed consent was obtained from the patient for publication of this case report and any accompanying images. A copy of the written consent is available for review by the Editor-in-Chief of this journal.

## Competing interests

The authors declare that they have no any competing interests.

## Authors' contributions

JE and MT contributed to the acquisition of data and interpreted experiments. KG, RM, HN and MMS interpreted experiments and revised the manuscript. All authors read and approved the final manuscript.
